# Xylopyranose Ring‐Opening by Single and Double Proton Transfers Under Pyrolysis Conditions

**DOI:** 10.1002/jcc.70151

**Published:** 2025-06-13

**Authors:** Jacopo Lupi, Bernardo Ballotta, Leandro Ayarde‐Henríquez, Stephen Dooley

**Affiliations:** ^1^ CNR‐ICCOM Consiglio Nazionale Delle Ricerche Pisa Italy; ^2^ School of Physics Trinity College Dublin Dublin Ireland; ^3^ AMBER, Advance Materials and BioEngineering Research Centre Dublin Ireland

**Keywords:** automated reaction mechanism discovery, gas‐phase thermochemistry, hemicellulose, multi‐path canonical variational transition state theory (MP‐VTST), multi‐structural torsional method (MS‐T)

## Abstract

This study unveils a new transition state (TS) leading to the acyclic product via synchronous double proton transfer by automatedly exploring the potential energy surface of β‐D‐xylopyranose under pyrolysis conditions. Quantum chemistry methods with multi‐path canonical variational transition state theory show that the standard activation enthalpy of the new TS (44.9 kcal ${\text{mol}}^{-1}$) is 1.5 kcal ${\text{mol}}^{-1}$ lower than that of the well‐established channel; however, the latter's rate constant ($4.36\times 10^{-2}$–$9.96\times 10^{1}$ ${\text{s}}^{-1}$) is higher in the 673.15–873.15 K pyrolytic range by a factor of 5–8. This gap narrows to a factor of 2 within 320–400 K, signifying that the new TS can potentially impact the acyclic product production in this low‐temperature regime. This is particularly relevant for β‐D‐xylopyranose trimers, as the interior unit bears different substituents at the C1 and C3 positions.

## Graphical abstract

Using automated reaction discovery tools, quantum chemistry, and kinetic modeling, we identify and characterize a novel transition state for xylopyranose ring opening reaction. This finding enhances the molecular‐level understanding of hemicellulose pyrolysis, providing new insight into reaction pathways relevant to biomass conversion and informing kinetic models for engineering applications.

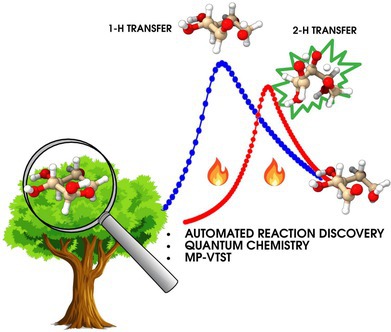

## 1 Introduction

**FIGURE 1:**
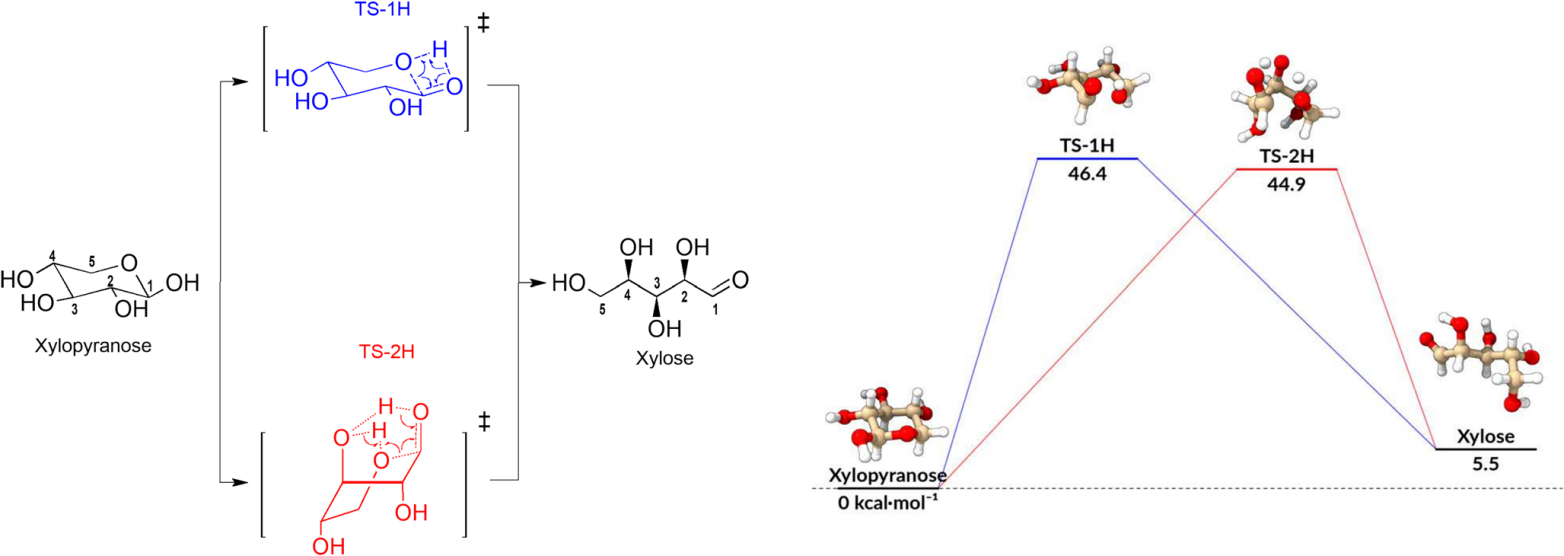
In the left panel: Single (TS‐1H) and double (TS‐2H) proton transfer mechanisms lead to the ring‐opening of xylopyranose. In the right panel: The ring‐opening barriers of both pathways, showing that the standard activation enthalpy of the new TS‐2H lies 1.5 kcal ${\text{mol}}^{-1}$ below. The new pathway is in red, and the known channel is in blue.

In recent decades, β‐D‐xylopyranose (hereafter termed xylopyranose), the hemicellulose building block, has received significant attention. Indeed, recent studies have focused on elucidating its structure, pyrolytic reactivity, and the effects of its functionalization [1, 2]. It is widely accepted in the literature that the predominant reaction pathway for xylopyranose thermal decomposition is the ring‐opening reaction [3]. This process is a concerted reaction involving a hydrogen transfer from the anomeric hydroxyl group to the ring oxygen, forming open‐chain D‐xylose (hereafter termed xylose). Recent high‐level electronic structure calculations and kinetic analysis have determined a standard activation enthalpy of 43.5–45.5 kcal ${\text{mol}}^{-1}$ for this elementary step, resulting in a faster reaction rate constant, 1.3–4.1 ${\text{s}}^{-1}$ at 773.15 K, compared to other potential initial reaction channels [1, 4]. To the best of the authors' knowledge, no alternative ring‐opening transition states (TSs) have been proposed until now for hemicellulose building blocks, whilst for cellulose ones, single, double, and triple proton transfer TSs were identified [5]. Utilizing state‐of‐the‐art automated reaction discovery codes (AutoMeKin [6]), we report a new reaction pathway leading to the formation of xylose, which undergoes a synchronous 2‐H proton transfer. This newly identified mechanism is illustrated in Figure 1, alongside the well‐known 1‐H proton transfer mechanism. Canonical thermal rate constants for both reactions are also computed employing multi‐path variational transition state theory (MP‐VTST).

## 2 Methods

### 2.1 Discovery of Xylopyranose Ring‐Opening Reaction Mechanisms

Reaction pathways for xylopyranose ring‐opening processes were generated using the AutoMeKin program [6, 7, 8], which is designed for automated reaction mechanism discovery. AutoMeKin employs methodologies rooted in graph theory, reactive molecular dynamics, and electronic structure calculations to fully explore potential energy surfaces (PESs), thereby facilitating the identification of potential reaction mechanisms.

The dynamic simulations required to obtain the initial TS structures were conducted with the semi‐empirical PM7 method [9] as implemented in the MOPAC package [10]. For these simulations, ten trajectories per iteration were calculated over a total of one hundred iterations. To screen and avoid redundant structures resulting from intermediate fragmentation, we applied specific criteria: the smallest accepted imaginary frequency was set at 100 ${\text{cm}}^{-1}$ (keyword imagmin), to account for torsional TSs, and the lowest eigenvalue of the Laplacian was set to 0.1 (keyword eigLmax). Additional screening parameters, such as MAPE (mean absolute percentage error) max and BAPE (biggest absolute percentage error) max, were set to 0.002 and 1.5, respectively, to compare descriptors characterizing the structures obtained from the molecular dynamics simulations. Further details on these parameters are provided in ref. [6]. More details about the resulting reaction network are provided in the Supporting Information.

Subsequently, optimized geometries and zero‐point corrected electronic energies for all PES' critical points, such as reactants, TSs, intermediates, and products, were determined using the revDSD‐PBEP86 [11] and D3(BJ) [12] double‐hybrid functional in combination with the jun‐cc‐pVTZ basis set [13], hereafter referred to as rDSD. Optimized Cartesian coordinates are reported in the Supporting Information.

The characterization of such structures along the reaction pathways as either minima (reactants, intermediates, products) or saddle points (TSs) was achieved through diagonalization of analytical rDSD Hessians. Moreover, intrinsic reaction coordinates (IRCs) [14] were traced starting from the identified TSs to characterize the elementary steps further and ensure the TSs connect the correct reactant and product. IRC plots are reported in Figure S1. All DFT calculations were performed using Gaussian16 [15].

The extensive and complex reaction networks generated by AutoMeKin were analyzed using the AMK tool [16], which facilitates the visualization of molecular structures, vibrational normal modes, and potential energy profiles of the investigated reaction mechanisms. Through this approach, we identified possible reaction pathways leading to xylopyranose ring‐opening and characterized the critical points governing these processes.

### 2.2 Energy Refinement

The computed electronic energies were subsequently refined by using the DLPNO‐CCSD(T) level of theory [17], with the F12 explicit correlation correction, on top of revDSD geometries. This method was selected due to its proven ability to deliver results that closely approximate those of the canonical CCSD(T) approach while significantly reducing the computational cost. The *tightPNO*cutoff setting was used to increase the accuracy of the localized pair natural orbital (PNO) approach, ensuring reliable results for the most complex systems. The choice of the cc‐pVTZ‐F12 basis set [18], provides an optimal balance between computational efficiency and accuracy, particularly when combined with explicitly correlated methods. All DLPNO calculations were performed using the ORCA quantum chemistry program [19]. The outcomes show that the new TS‐2H is energetically favorable as its standard activation enthalpy is approximately 1.5 kcal ${\text{mol}}^{-1}$ lower than the one characterizing the single proton transfer. See Figure 1, right panel.

### 2.3 Kinetics

Within VTST's framework, computing rate constants involves critical aspects, including variational effects and torsional anharmonicity, especially for systems with multiple conformers (or structures) of reactants and TSs. The program Pilgrim [20] enables the detailed computation of thermal rate constants considering these factors by gauging MP‐VTST and treating the torsional anharmonicity via subroutines implemented in the MSTor program [21].

For a reaction proceeding from a reactant to a product through a TS, the canonical rate constant k(T) at the absolute temperature T is given by the Eyring equation within the framework of conventional transition state theory (TST):

(1) $$k(T)=\kappa (T)\frac{k_{B}T}{h}\frac{Q^{‡}(T)}{Q_{R}(T)}\exp\left(-\frac{\Delta E^{‡}}{RT}\right)$$

where R is the universal constant of the ideal gas, $k_{B}$ is the Boltzmann constant, h is Planck's constant, $Q^{‡}(T)$ and $Q_{R}(T)$ are the partition functions of the TS and reactant, respectively, $\Delta E^{‡}$ is the potential energy barrier height, and κ(T) is the transmission coefficient that accounts for quantum tunneling effects.

In VTST, the location of the dividing surface is varied to minimize the rate constant, leading to the canonical variational transition state theory (CVT) expression:

(2) $$k_{\text{CVT}}(T)=\underset{s}{\min}\left\{\kappa (T,s)\frac{k_{B}T}{h}\frac{Q^{‡}(T,s)}{Q_{R}(T)}\exp\left(-\frac{V(s)}{RT}\right)\right\}$$

where the reaction coordinate s is varied to find the minimum value of the rate constant, V(s) is the potential energy, and κ(T,s) is the temperature‐dependent transmission coefficient.

In reactions with multiple possible transition states or pathways, MP‐VTST provides a more accurate estimation of the overall reaction rate by summing the contributions of each pathway, weighted by their individual rate constants.

The overall rate constant $k_{\text{MP‐CVT}}$ is given by:

(3) $$k_{\text{MP‐CVT}}(T)=\underset{j}{\sum }k_{j}(T)$$

where $k_{j}(T)$ is the rate constant of the jth reaction path.

Quantum mechanical tunneling effects have been considered by using small curvature tunneling (SCT) corrections. The transmission coefficient $\kappa_{j}$ of each path is given by:

(4) $$\kappa_{j}=\exp\left(-\frac{2}{\hbar }\int_{s_{1}}^{s_{2}}{\left[2\mu \left(V(s)-E\right)\right]}^{1/2}ds\right)$$

where μ is the reduced mass along the reaction coordinate, $s_{1}$ and $s_{2}$ are the turning points of the reaction coordinate where V(s)=E.

By summing the rate constants for all significant pathways, MP‐VTST provides a comprehensive rate constant that accounts for the contributions of multiple reaction mechanisms, each characterized by its own TS and PES. This method is particularly useful for complex reactions with competing pathways, ensuring a more accurate prediction of the overall reaction kinetics.

Torsional anharmonicity, arising from the non‐rigid nature of molecular torsional modes, can significantly affect the partition functions and, consequently, the rate constants. For each mode, the torsional anharmonic partition function, $Q_{\text{tor}}$, is computed using the MSTor program:

(5) $$Q_{\text{tor}}=\int_{0}^{2\pi }\exp\left(-\frac{V(\phi )}{k_{B}T}\right)d\phi$$

where V(ϕ) is the potential energy as a function of the torsional angle ϕ. See the Supporting Information for detailed analyses of anharmonicity, tunneling, recrossing coefficients, the total partition functions, and a comparison of transition state theories.

**FIGURE 2:**
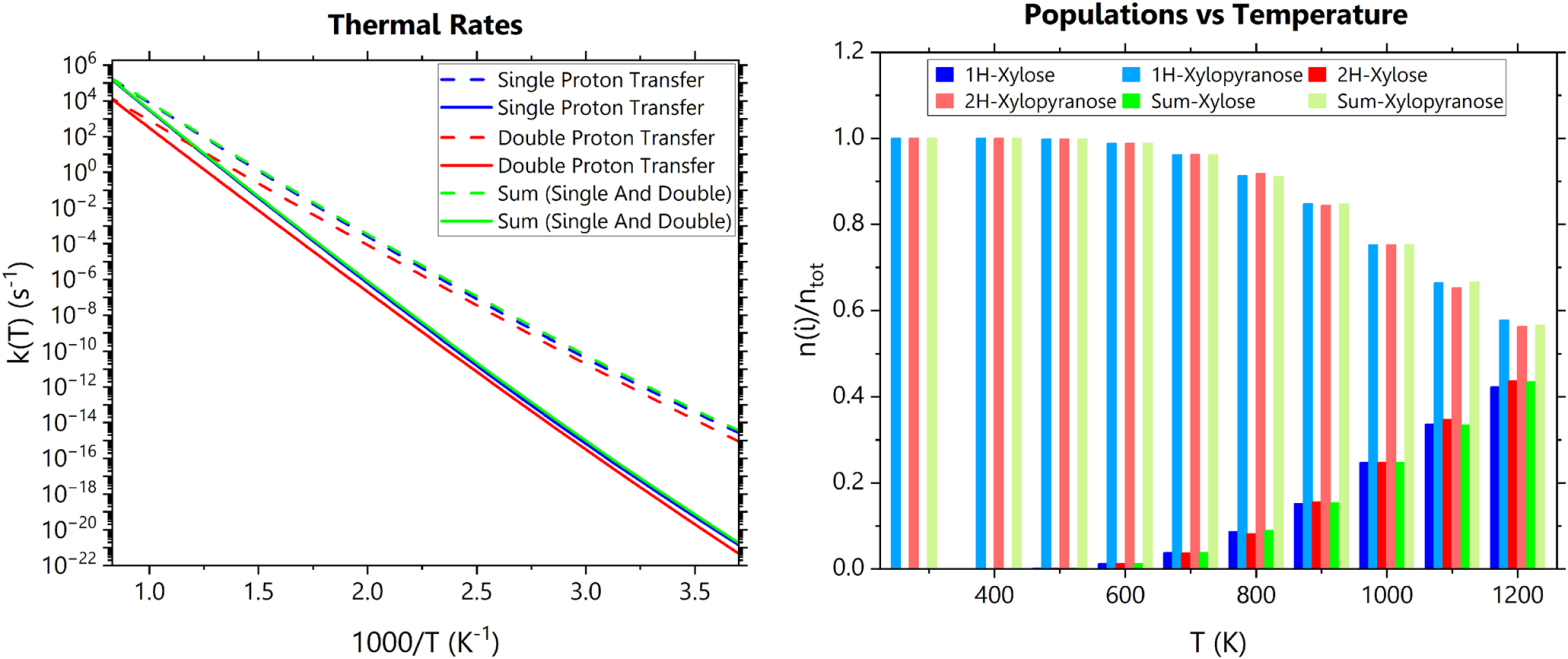
The left panel depicts the rate constants of the xylopyranose→xylose (solid lines) and xylose→xylopyranose reactions (dashed lines) for the single and double proton transfers, along with the sum of both channels' kinetics (green lines). In the right panel, a kinetic Monte Carlo simulation shows that, for both mechanisms, the relative population of xylose becomes significant for temperatures higher than the upper limit of the pyrolysis regime (approximately 880 K).

The kinetic results unveil that the single proton transfer rate exceeds that of the new TS in both the forward (xylopyranose→xylose) and backward (xylose→xylopyranose) directions across the temperature range. In particular, the rate ratio of the ring‐opening process ranges from five to eight within the pyrolysis regime, 673.15–873.15 K. Additionally, at any given temperature, both mechanisms are kinetically faster in the backward direction, highlighting their thermally driven nature (see Figure 2, left panel). For single and double proton migrations, kinetic Monte Carlo (kMC) simulations show that the xylopyranose‐to‐xylose thermal conversion becomes significant for temperatures higher than those characterizing the pyrolysis range (see Figure 2, right panel). The Arrhenius parameters derived from fitting the forward rates of the single proton channel align closely with recent reports [1, 4], as presented in Table 1. A quantitative analysis of the thermal rate constants for both mechanisms over a temperature range relevant to pyrolysis is presented in the Supporting Information. Specifically, Figures S2 and S3 display plots of anharmonicity, recrossing, tunneling, and transmission coefficients, while Figure S4 shows the Arrhenius plots of the rate constants. Numerical values of the rate constants and partition functions across the studied temperature range are provided in Tables S1 and S2. Additional details on the kinetic Monte Carlo (kMC) simulations are also included in the Supporting Information.

**TABLE 1:** Fitted Arrhenius parameters over the 270–1200 K temperature range for the single (1H‐PT) and double (2H‐PT) proton transfer.

	MP‐CVT 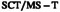	1H‐PT‐CVT 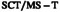	2H‐PT‐CVT 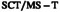
A/${\text{s}}^{-1}$	$3.16\times 10^{13}$/$5.28\times 10^{11}$	$3.58\times 10^{13}$/$6.92\times 10^{11}$	$7.82\times 10^{11}$/$1.28\times 10^{10}$
$E_{a}$/kcal ${\text{mol}}^{-1}$	45.7/35.7	46.2/36.5	43.0/33.0
RMSE/${\text{s}}^{-1}$	223.39/57.66	179.75/450.31	3.14/5.49

*Note:* The xylopyranose→xylose/xylose→xylopyranose reaction data and associated root mean square error (RMSE) are reported in each entry. CVT is the canonical variational transition state theory, SCT stands for semiclassical small‐curvature tunneling approximation, and MS‐T corresponds to the multi‐structural torsional method.

This study reveals a novel TS for the ring opening of β‐D‐xylopyranose via synchronous double proton transfer, with a 1.5 kcal ${\text{mol}}^{-1}$ lower activation enthalpy than the established single proton transfer pathway. Despite its negligible kinetic contribution to high‐temperature pyrolysis, the new TS may influence reaction dynamics at lower temperatures. These findings underscore the importance of exploring alternative pathways through computational techniques to enhance kinetic models for the thermal decomposition of biomass‐derived carbohydrates.

## Conflicts of Interest

The authors declare no conflicts of interest.

## Supporting information


**Data S1.** Supporting Information.

## Data Availability

The data that supports the findings of this study are available in the Supporting Information of this article.
