# Supplementary material for: Xylopyranose Ring‐Opening by Single and Double Proton Transfers Under Pyrolysis Conditions
Source: J Comput Chem. 2025 Jun 13;46(16):e70151. doi: 10.1002/jcc.70151 (PMC12163559; doi:10.1002/jcc.70151)
Supplement: Supplementary file 1 — Data S1. Supporting Information. [file JCC-46-0-s001.pdf]

**Supplementary Material**

# Xylopyranose ring-opening by single and double proton transfers under pyrolysis conditions

Jacopo Lupi,*[1] Bernardo Ballotta,*[2,3] Leandro Ayarde-Henríquez,[2,3] and Stephen Dooley[2,3]

[1]CNR-ICCOM, Consiglio Nazionale delle Ricerche, Via Giuseppe Moruzzi 1 Pisa-56124, Italy.
[2]School of Physics, Trinity College Dublin, Dublin 2, Ireland.
[3]AMBER, Advanced Materials and BioEngineering Research Centre, Dublin 2, Ireland.

Correspondence to: jacopo.lupi@cnr.it
bernardo.ballotta@tcd.ie

## Contents

**1. Optimized Cartesian coordinates for minima and TSs of β-D-xylopyranose's ring-opening mechanisms at the revDSD-PBEP86/jun-cc-pVTZ level.** ........ S1

**2. Intrinsic reaction coordinates plots for both proton transfer mechanisms.** ........ S2

**3. Coefficients derived from multi-path and -structural canonical variational transition state theories.** ........ S3

**4. Eckart coefficients.** ........ S4

**5. Canonical transition state vs canonical variational transition state theories.** ........ S4

**Table S1. Computed thermal rates of ring-opening for both single (1H-PT) and double (2H-PT) proton transfers in the 270-1200 K temperature range at the CVT$^{SCT/MS-T}$ level.** ........ S5

**Table S2. Total partition functions of β-D-xylopyranose, D-xylose, and single (1H-PT) and double (2H-PT) proton transfer transition states in the 270-1200 K temperature range, including the electronic, translational, and rovibrational components.** ........ S6

**6. AutoMeKin reaction network details** ........ S7

**7. Kinetic Monte Carlo simulation details** ........ S7

## 1. Optimized Cartesian coordinates for minima and TSs of β-D-xylopyranose's ring-opening mechanisms at the revDSD-PBEP86-D3(BJ)/jun-cc-pVTZ level.

Reactant (β-D-xylopyranose)

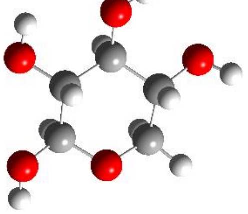

| | | | |
|---|---|---|---|
| C | -1.410836 | -0.527094 | -0.290443 |
| C | -0.605226 | -1.731418 | 0.178986 |
| C | 1.394319 | -0.509243 | 0.248833 |
| C | 0.712378 | 0.767551 | -0.223867 |
| C | -0.743843 | 0.745507 | 0.199589 |
| H | -0.637778 | -1.787255 | 1.275378 |
| H | -1.018563 | -2.651862 | -0.228964 |
| H | -3.183775 | 0.165799 | 0.042496 |
| H | 1.374540 | -0.561732 | 1.349643 |
| H | 0.780046 | 0.796245 | -1.317985 |
| H | -0.788417 | 0.761448 | 1.297856 |
| O | 0.738742 | -1.642454 | -0.284252 |
| O | 2.702831 | -0.479123 | -0.230890 |
| H | 3.163511 | -1.261246 | 0.090564 |
| O | 1.297651 | 1.924715 | 0.354248 |
| H | 2.222806 | 1.947274 | 0.086205 |
| O | -1.471280 | 1.843523 | -0.331466 |
| H | -1.013522 | 2.648498 | -0.064273 |
| O | -2.722214 | -0.659775 | 0.228842 |
| H | -1.427046 | -0.511465 | -1.387503 |

Product (D-xylose)

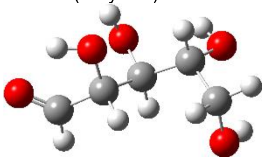

| | | | |
|---|---|---|---|
| C | 1.32128900 | 0.38395800 | 0.48433900 |
| C | 2.04206800 | -0.95012400 | 0.57516700 |
| C | -2.22321600 | -0.61133600 | -0.72512400 |
| C | -1.04996800 | -0.52733700 | 0.22801800 |
| C | 0.05203900 | 0.34399900 | -0.36882000 |
| H | 2.94884400 | -0.80793400 | 1.17300400 |
| H | 1.42207200 | -1.69291700 | 1.07846600 |
| H | 1.05848000 | 0.71653000 | 1.49733200 |
| H | -2.00125500 | -0.94237500 | -1.75366000 |
| H | -0.67034500 | -1.55195100 | 0.35240100 |
| H | 0.31300200 | -0.03068300 | -1.36086400 |
| O | 2.35349900 | -1.47285500 | -0.70727100 |
| O | -3.34883000 | -0.34328300 | -0.36842400 |
| H | 2.87443900 | -0.79689300 | -1.15621800 |
| O | -1.46580500 | 0.01489900 | 1.46098600 |
| H | -2.43582800 | 0.02964500 | 1.43058000 |
| O | -0.43552300 | 1.67118700 | -0.55655600 |
| H | -0.87541600 | 1.91679600 | 0.26891000 |
| O | 2.24448400 | 1.28967500 | -0.11297100 |
| H | 1.73014100 | 2.04784000 | -0.41754000 |

Transition state of the single-proton transfer (TS-1H)

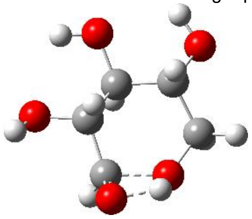

| | | | |
|---|---|---|---|
| C | 1.65898200 | -0.27800200 | 0.28148400 |
| O | 0.77370400 | -1.67497100 | 0.30168500 |
| H | 1.57394900 | -1.73515000 | -0.52979000 |
| O | 2.52021300 | -0.77331000 | -0.57508700 |
| C | -1.30362100 | -0.53892400 | -0.34496100 |
| C | -0.65994000 | -1.78487400 | 0.24172700 |
| C | 0.75062800 | 0.85453200 | -0.19711200 |
| C | -0.68710000 | 0.71161000 | 0.26436800 |
| H | -1.02380800 | -1.92183100 | 1.26137000 |
| H | -0.89954900 | -2.67256500 | -0.34236400 |
| H | -1.14887200 | -0.50056800 | -1.43176200 |
| H | 1.96107200 | -0.15849400 | 1.32990300 |
| H | 0.77551100 | 0.85747200 | -1.29193900 |
| H | -0.72490100 | 0.64456500 | 1.36117200 |
| O | 1.22581300 | 2.09643200 | 0.31748100 |
| H | 2.05137800 | 2.31315000 | -0.12850900 |
| O | -1.47022000 | 1.80633500 | -0.18560600 |
| H | -0.98753600 | 2.61098000 | 0.03773700 |
| O | -2.68211600 | -0.63575200 | -0.04602400 |
| H | -3.07008400 | 0.22652200 | -0.23844600 |

Transition state of the double-proton transfer (TS-2H)

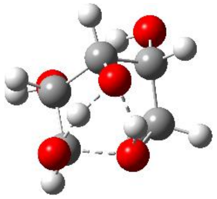

| | | | |
|---|---|---|---|
| C | -1.35813700 | -0.71756900 | -0.21059200 |
| C | -0.78689100 | -0.84326800 | 1.21652100 |
| C | 1.42906100 | 0.50977500 | 0.56666500 |
| C | 0.38308800 | 1.09865300 | -0.38324300 |
| C | -0.25428000 | -0.11542100 | -1.10172400 |
| H | -1.04157500 | 0.03109400 | 1.81266200 |
| H | -1.12584300 | -1.74288000 | 1.72873000 |
| H | -1.57129600 | -1.70689700 | -0.61764700 |
| H | 1.60257900 | 1.01240500 | 1.51904700 |
| H | 0.94399800 | 1.70420500 | -1.09912300 |
| H | -0.67741700 | 0.17815800 | -2.06513700 |
| O | 0.66567900 | -0.92147400 | 1.13067400 |
| O | 2.46275500 | 0.05689300 | -0.08603800 |
| H | 1.84251300 | -0.53878600 | -0.90044600 |
| O | -0.62117500 | 1.88562800 | 0.25508900 |
| H | -0.30431300 | 2.78912500 | 0.33798300 |
| O | 0.75673200 | -1.11305600 | -1.24912400 |
| H | 0.79516400 | -1.35374900 | 0.13189200 |
| O | -2.58367100 | -0.01601700 | -0.22364900 |
| H | -2.38342300 | 0.89851300 | 0.01065500 |

## 2. Intrinsic reaction coordinates plots for both proton transfer mechanisms.

(a) (b)

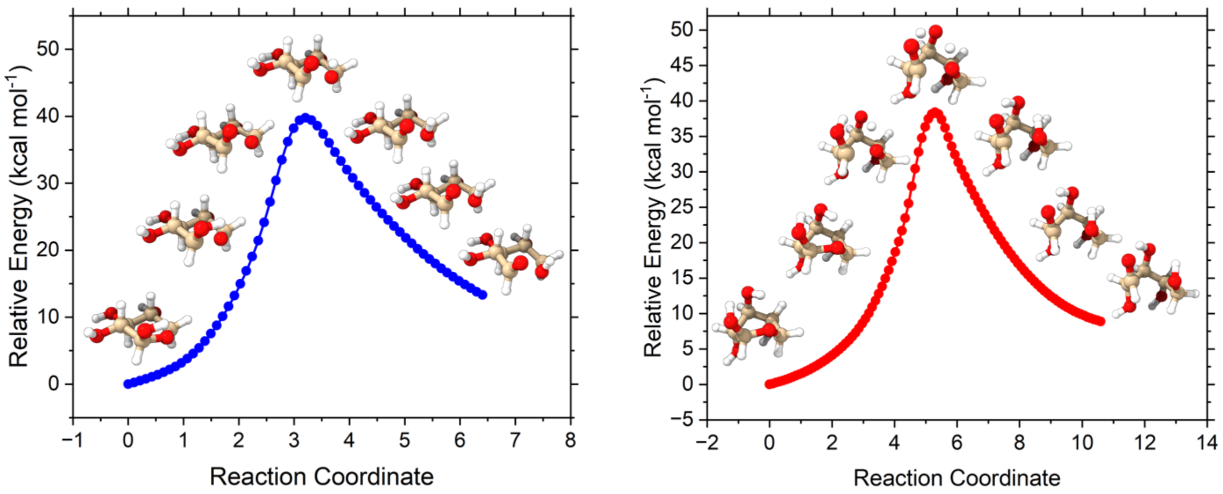


**Figure S1.** Intrinsic reaction coordinate of the single (panel a) and double (panel b) proton transfers.

## 3. Coefficients derived from multi-path and -structural canonical variational transition state theories.

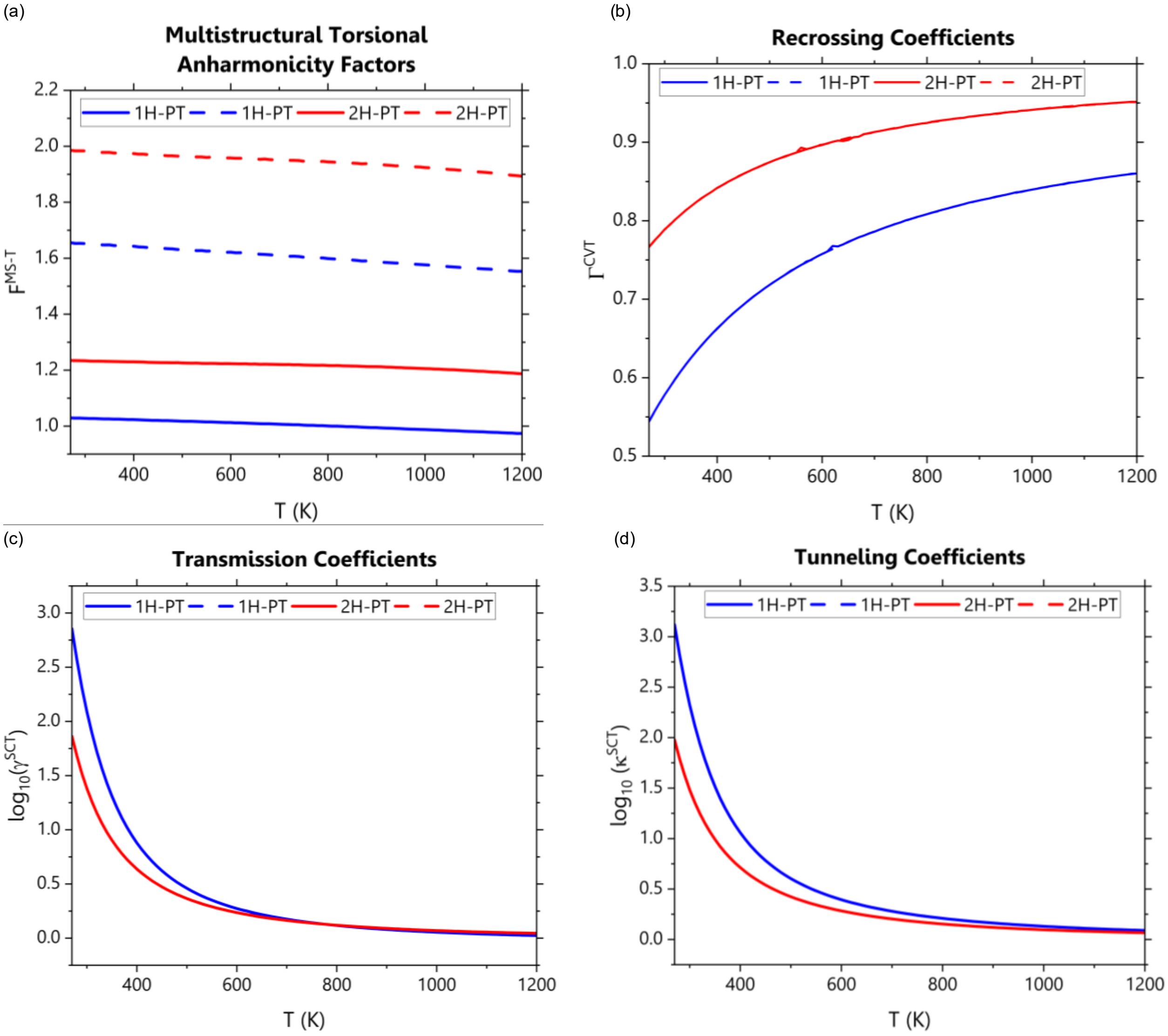


**Figure S2.** Anharmonic (panel a), recrossing (panel b), transmission (panel c), and tunneling coefficients (panel d) as functions of temperature for the single (1H-PT) and double (2H-PT) proton transfer mechanisms. Solid and dashed lines indicate the forward (β-D-xylopyranose→D-xylose) and backward (D-xylose→β-D-xylopyranose) directions, respectively.

## 4. Eckart coefficients.

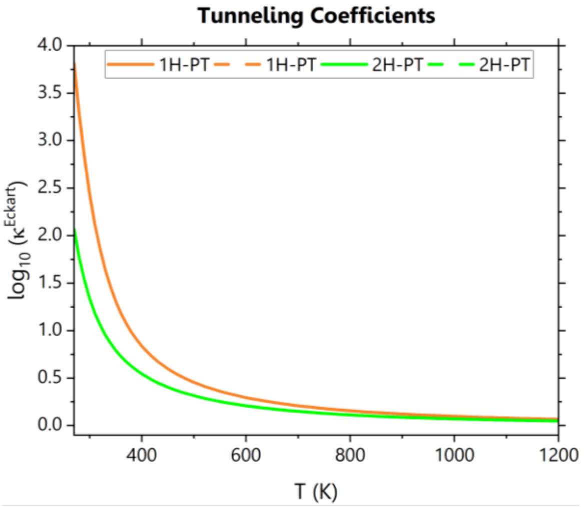


**Figure S3.** Eckart tunneling coefficients as functions of temperature for the single (1H-PT) and double (2H-PT) proton transfer mechanisms. Solid and dashed lines indicate the forward (β-D-xylopyranose→D-xylose) and backward (D-xylose→β-D-xylopyranose) directions, respectively.

## 5. Canonical transition state vs canonical variational transition state theories.

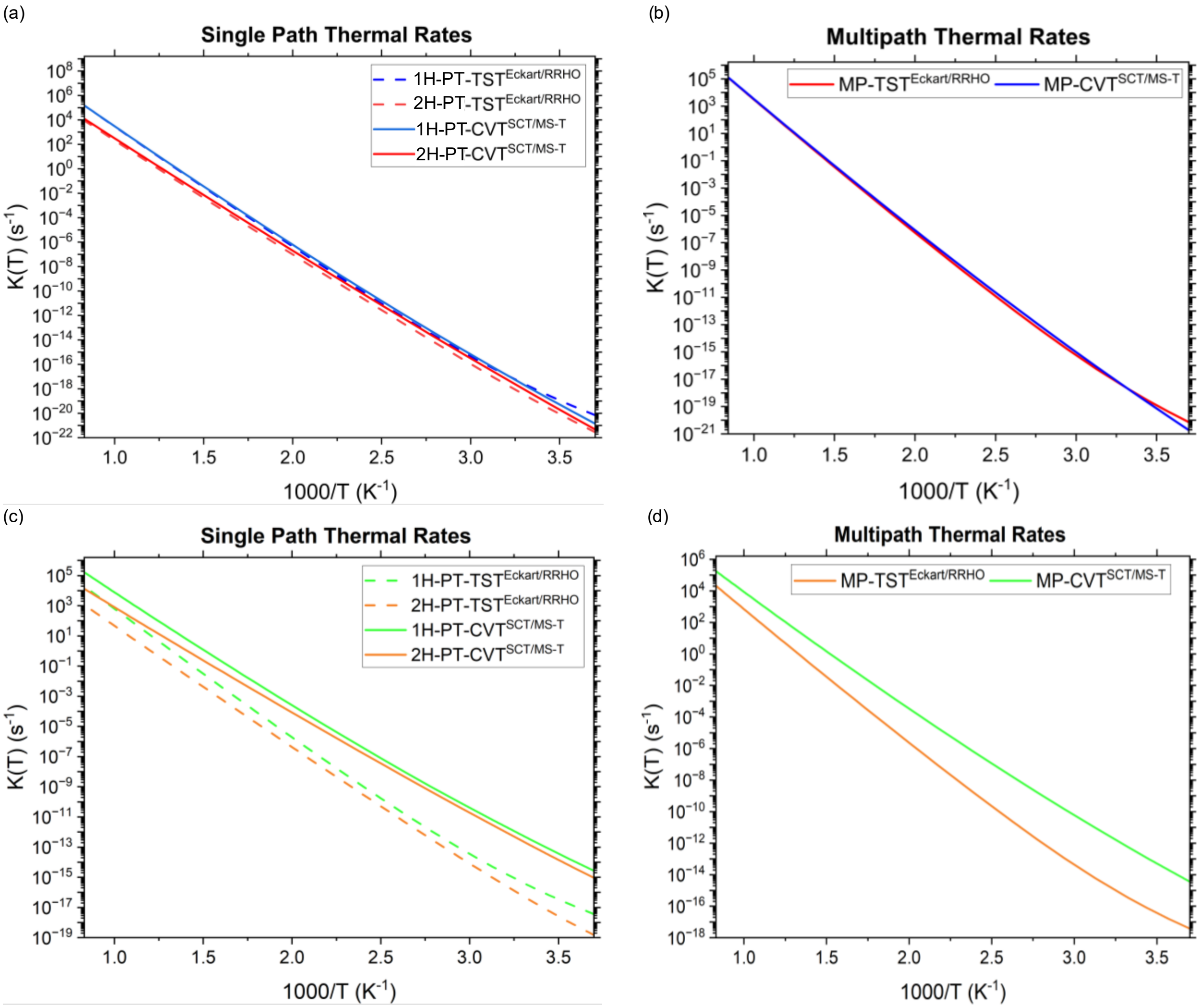


**Figure S4.** Computed rate constants of the single (1H-PT) and double (2H-PT) proton transfers in the β-D-xylopyranose→D-xylose (Panels a-b) and D-xylose→β-D-xylopyranose (Panels c-d) processes.

**Table S1.** Computed thermal rates of ring-opening for both single (1H-PT) and double (2H-PT) proton transfers in the 270-1200 K temperature range at the $CVT^{SCT/MS-T}$ level.

| T /K | 1H-PT | 2H-PT | 1:2 /% | T /K | 1H-PT | 2H-PT | 1:2 /% | T /K | 1H-PT | 2H-PT | 1:2 /% |
|---|---|---|---|---|---|---|---|---|---|---|---|
| 270 | $1.31 \times 10^{-21}$ | $4.35 \times 10^{-22}$ | 3.00 | 590 | $4.86 \times 10^{-4}$ | $1.19 \times 10^{-4}$ | 4.07 | 910 | $3.15 \times 10^{2}$ | $3.71 \times 10^{1}$ | 8.48 |
| 280 | $1.41 \times 10^{-20}$ | $5.15 \times 10^{-21}$ | 2.73 | 600 | $9.08 \times 10^{-4}$ | $2.16 \times 10^{-4}$ | 4.20 | 920 | $4.13 \times 10^{2}$ | $4.80 \times 10^{1}$ | 8.61 |
| 290 | $1.33 \times 10^{-19}$ | $5.28 \times 10^{-20}$ | 2.53 | 610 | $1.66 \times 10^{-3}$ | $3.84 \times 10^{-4}$ | 4.33 | 930 | $5.39 \times 10^{2}$ | $6.17 \times 10^{1}$ | 8.75 |
| 300 | $1.13 \times 10^{-18}$ | $4.74 \times 10^{-19}$ | 2.37 | 620 | $2.99 \times 10^{-3}$ | $6.70 \times 10^{-4}$ | 4.46 | 940 | $7.00 \times 10^{2}$ | $7.88 \times 10^{1}$ | 8.89 |
| 310 | $8.54 \times 10^{-18}$ | $3.77 \times 10^{-18}$ | 2.26 | 630 | $5.28 \times 10^{-3}$ | $1.15 \times 10^{-3}$ | 4.59 | 950 | $9.04 \times 10^{2}$ | $1.00 \times 10^{2}$ | 9.03 |
| 320 | $5.85 \times 10^{-17}$ | $2.68 \times 10^{-17}$ | 2.18 | 640 | $9.16 \times 10^{-3}$ | $1.94 \times 10^{-3}$ | 4.72 | 960 | $1.16 \times 10^{3}$ | $1.27 \times 10^{2}$ | 9.16 |
| 330 | $3.65 \times 10^{-16}$ | $1.72 \times 10^{-16}$ | 2.13 | 650 | $1.57 \times 10^{-2}$ | $3.22 \times 10^{-3}$ | 4.86 | 970 | $1.49 \times 10^{3}$ | $1.60 \times 10^{2}$ | 9.29 |
| 340 | $2.09 \times 10^{-15}$ | $9.96 \times 10^{-16}$ | 2.10 | 660 | $2.63 \times 10^{-2}$ | $5.27 \times 10^{-3}$ | 4.99 | 980 | $1.89 \times 10^{3}$ | $2.00 \times 10^{2}$ | 9.43 |
| 350 | $1.10 \times 10^{-14}$ | $5.28 \times 10^{-15}$ | 2.08 | 670 | $4.36 \times 10^{-2}$ | $8.50 \times 10^{-3}$ | 5.13 | 990 | $2.39 \times 10^{3}$ | $2.50 \times 10^{2}$ | 9.56 |
| 360 | $5.36 \times 10^{-14}$ | $2.57 \times 10^{-14}$ | 2.09 | 680 | $7.12 \times 10^{-2}$ | $1.35 \times 10^{-2}$ | 5.26 | 1000 | $3.01 \times 10^{3}$ | $3.11 \times 10^{2}$ | 9.69 |
| 370 | $2.44 \times 10^{-13}$ | $1.16 \times 10^{-13}$ | 2.10 | 690 | $1.15 \times 10^{-1}$ | $2.12 \times 10^{-2}$ | 5.40 | 1010 | $3.78 \times 10^{3}$ | $3.85 \times 10^{2}$ | 9.83 |
| 380 | $1.03 \times 10^{-12}$ | $4.85 \times 10^{-13}$ | 2.13 | 700 | $1.83 \times 10^{-1}$ | $3.29 \times 10^{-2}$ | 5.54 | 1020 | $4.72 \times 10^{3}$ | $4.74 \times 10^{2}$ | 9.96 |
| 390 | $4.12 \times 10^{-12}$ | $1.90 \times 10^{-12}$ | 2.17 | 710 | $2.87 \times 10^{-1}$ | $5.05 \times 10^{-2}$ | 5.68 | 1030 | $5.88 \times 10^{3}$ | $5.82 \times 10^{2}$ | $1.01 \times 10^{1}$ |
| 400 | $1.54 \times 10^{-11}$ | $6.97 \times 10^{-12}$ | 2.21 | 720 | $4.45 \times 10^{-1}$ | $7.64 \times 10^{-2}$ | 5.82 | 1040 | $7.28 \times 10^{3}$ | $7.12 \times 10^{2}$ | $1.02 \times 10^{1}$ |
| 410 | $5.47 \times 10^{-11}$ | $2.41 \times 10^{-11}$ | 2.27 | 730 | $6.82 \times 10^{-1}$ | $1.15 \times 10^{-1}$ | 5.95 | 1050 | $8.98 \times 10^{3}$ | $8.67 \times 10^{2}$ | $1.04 \times 10^{1}$ |
| 420 | $1.84 \times 10^{-10}$ | $7.90 \times 10^{-11}$ | 2.33 | 740 | 1.03 | $1.70 \times 10^{-1}$ | 6.10 | 1060 | $1.10 \times 10^{4}$ | $1.05 \times 10^{3}$ | $1.05 \times 10^{1}$ |
| 430 | $5.89 \times 10^{-10}$ | $2.45 \times 10^{-10}$ | 2.40 | 750 | 1.55 | $2.49 \times 10^{-1}$ | 6.24 | 1070 | $1.35 \times 10^{4}$ | $1.27 \times 10^{3}$ | $1.06 \times 10^{1}$ |
| 440 | $1.80 \times 10^{-9}$ | $7.26 \times 10^{-10}$ | 2.48 | 760 | 2.30 | $3.61 \times 10^{-1}$ | 6.38 | 1080 | $1.65 \times 10^{4}$ | $1.54 \times 10^{3}$ | $1.07 \times 10^{1}$ |
| 450 | $5.24 \times 10^{-9}$ | $2.05 \times 10^{-9}$ | 2.56 | 770 | 3.39 | $5.20 \times 10^{-1}$ | 6.52 | 1090 | $2.00 \times 10^{4}$ | $1.84 \times 10^{3}$ | $1.09 \times 10^{1}$ |
| 460 | $1.47 \times 10^{-8}$ | $5.55 \times 10^{-9}$ | 2.64 | 780 | 4.93 | $7.41 \times 10^{-1}$ | 6.66 | 1100 | $2.43 \times 10^{4}$ | $2.21 \times 10^{3}$ | $1.10 \times 10^{1}$ |
| 470 | $3.94 \times 10^{-8}$ | $1.44 \times 10^{-8}$ | 2.73 | 790 | 7.12 | 1.05 | 6.80 | 1110 | $2.93 \times 10^{4}$ | $2.64 \times 10^{3}$ | $1.11 \times 10^{1}$ |
| 480 | $1.02 \times 10^{-7}$ | $3.61 \times 10^{-8}$ | 2.83 | 800 | $1.02 \times 10^{1}$ | 1.47 | 6.94 | 1120 | $3.53 \times 10^{4}$ | $3.14 \times 10^{3}$ | $1.13 \times 10^{1}$ |
| 490 | $2.55 \times 10^{-7}$ | $8.72 \times 10^{-8}$ | 2.93 | 810 | $1.44 \times 10^{1}$ | 2.04 | 7.08 | 1130 | $4.23 \times 10^{4}$ | $3.72 \times 10^{3}$ | $1.14 \times 10^{1}$ |
| 500 | $6.16 \times 10^{-7}$ | $2.04 \times 10^{-7}$ | 3.03 | 820 | $2.03 \times 10^{1}$ | 2.81 | 7.22 | 1140 | $5.06 \times 10^{4}$ | $4.40 \times 10^{3}$ | $1.15 \times 10^{1}$ |
| 510 | $1.44 \times 10^{-6}$ | $4.60 \times 10^{-7}$ | 3.13 | 830 | $2.83 \times 10^{1}$ | 3.85 | 7.36 | 1150 | $6.03 \times 10^{4}$ | $5.19 \times 10^{3}$ | $1.16 \times 10^{1}$ |
| 520 | $3.27 \times 10^{-6}$ | $1.01 \times 10^{-6}$ | 3.24 | 840 | $3.92 \times 10^{1}$ | 5.23 | 7.50 | 1160 | $7.17 \times 10^{4}$ | $6.10 \times 10^{3}$ | $1.17 \times 10^{1}$ |
| 530 | $7.21 \times 10^{-6}$ | $2.15 \times 10^{-6}$ | 3.35 | 850 | $5.39 \times 10^{1}$ | 7.05 | 7.64 | 1170 | $8.50 \times 10^{4}$ | $7.16 \times 10^{3}$ | $1.19 \times 10^{1}$ |
| 540 | $1.55 \times 10^{-5}$ | $4.46 \times 10^{-6}$ | 3.47 | 860 | $7.35 \times 10^{1}$ | 9.45 | 7.78 | 1180 | $1.00 \times 10^{5}$ | $8.37 \times 10^{3}$ | $1.20 \times 10^{1}$ |
| 550 | $3.23 \times 10^{-5}$ | $9.01 \times 10^{-6}$ | 3.58 | 870 | $9.96 \times 10^{1}$ | $1.26 \times 10^{1}$ | 7.92 | 1190 | $1.18 \times 10^{5}$ | $9.77 \times 10^{3}$ | $1.21 \times 10^{1}$ |
| 560 | $6.59 \times 10^{-5}$ | $1.78 \times 10^{-5}$ | 3.70 | 880 | $1.34 \times 10^{2}$ | $1.66 \times 10^{1}$ | 8.06 | 1200 | $1.39 \times 10^{5}$ | $1.14 \times 10^{4}$ | $1.22 \times 10^{1}$ |
| 570 | $1.31 \times 10^{-4}$ | $3.43 \times 10^{-5}$ | 3.82 | 890 | $1.79 \times 10^{2}$ | $2.19 \times 10^{1}$ | 8.20 | | | | |
| 580 | $2.55 \times 10^{-4}$ | $6.47 \times 10^{-5}$ | 3.95 | 900 | $2.38 \times 10^{2}$ | $2.86 \times 10^{1}$ | 8.34 | | | | |

**Table S2.** Total partition functions of β-D-xylopyranose, D-xylose, and single (1H-PT) and double (2H-PT) proton transfer transition states in the 270-1200 K temperature range, including the electronic, translational, and rovibrational components.

| T /K | β-D-xylopyranose | D-xylose | 1H-PT | 2H-PT | T /K | β-D-xylopyranose | D-xylose | 1H-PT | 2H-PT |
|---|---|---|---|---|---|---|---|---|---|
| 270 | $1.4700 \times 10^{-67}$ | $2.8607 \times 10^{-65}$ | $2.5644 \times 10^{-64}$ | $3.9139 \times 10^{-65}$ | 740 | $4.7607 \times 10^{-8}$ | $3.7297 \times 10^{-6}$ | $7.1136 \times 10^{-7}$ | $3.8578 \times 10^{-8}$ |
| 280 | $1.9242 \times 10^{-64}$ | $3.5138 \times 10^{-62}$ | $2.5911 \times 10^{-61}$ | $3.8047 \times 10^{-62}$ | 750 | $1.6385 \times 10^{-7}$ | $1.2778 \times 10^{-5}$ | $2.3542 \times 10^{-6}$ | $1.2627 \times 10^{-7}$ |
| 290 | $1.5731 \times 10^{-61}$ | $2.7100 \times 10^{-59}$ | $1.6638 \times 10^{-58}$ | $2.3528 \times 10^{-59}$ | 760 | $5.4965 \times 10^{-7}$ | $4.2677 \times 10^{-5}$ | $7.6010 \times 10^{-6}$ | $4.0329 \times 10^{-7}$ |
| 300 | $8.4182 \times 10^{-59}$ | $1.3745 \times 10^{-56}$ | $7.1029 \times 10^{-56}$ | $9.6833 \times 10^{-57}$ | 770 | $1.7988 \times 10^{-6}$ | $1.3907 \times 10^{-4}$ | $2.3963 \times 10^{-5}$ | $1.2581 \times 10^{-6}$ |
| 310 | $3.0715 \times 10^{-56}$ | $4.7739 \times 10^{-54}$ | $2.0970 \times 10^{-53}$ | $2.7589 \times 10^{-54}$ | 780 | $5.7483 \times 10^{-6}$ | $4.4260 \times 10^{-4}$ | $7.3834 \times 10^{-5}$ | $3.8366 \times 10^{-6}$ |
| 320 | $7.9196 \times 10^{-54}$ | $1.1762 \times 10^{-51}$ | $4.4317 \times 10^{-51}$ | $5.6319 \times 10^{-52}$ | 790 | $1.7953 \times 10^{-5}$ | $1.3768 \times 10^{-3}$ | $2.2252 \times 10^{-4}$ | $1.1447 \times 10^{-5}$ |
| 330 | $1.4891 \times 10^{-51}$ | $2.1205 \times 10^{-49}$ | $6.9098 \times 10^{-49}$ | $8.4906 \times 10^{-50}$ | 800 | $5.4841 \times 10^{-5}$ | $4.1897 \times 10^{-3}$ | $6.5645 \times 10^{-4}$ | $3.3439 \times 10^{-5}$ |
| 340 | $2.0993 \times 10^{-49}$ | $2.8752 \times 10^{-47}$ | $8.1641 \times 10^{-47}$ | $9.7090 \times 10^{-48}$ | 810 | $1.6398 \times 10^{-4}$ | $1.2481 \times 10^{-2}$ | $1.8972 \times 10^{-3}$ | $9.5714 \times 10^{-5}$ |
| 350 | $2.2740 \times 10^{-47}$ | $3.0041 \times 10^{-45}$ | $7.4848 \times 10^{-45}$ | $8.6223 \times 10^{-46}$ | 820 | $4.8034 \times 10^{-4}$ | $3.6429 \times 10^{-2}$ | $5.3750 \times 10^{-3}$ | $2.6864 \times 10^{-4}$ |
| 360 | $1.9347 \times 10^{-45}$ | $2.4714 \times 10^{-43}$ | $5.4376 \times 10^{-43}$ | $6.0733 \times 10^{-44}$ | 830 | $1.3793 \times 10^{-3}$ | $1.0424 \times 10^{-1}$ | $1.4939 \times 10^{-2}$ | $7.3981 \times 10^{-4}$ |
| 370 | $1.3181 \times 10^{-43}$ | $1.6320 \times 10^{-41}$ | $3.1897 \times 10^{-41}$ | $3.4571 \times 10^{-42}$ | 840 | $3.8850 \times 10^{-3}$ | $2.9263 \times 10^{-1}$ | $4.0757 \times 10^{-2}$ | $2.0003 \times 10^{-3}$ |
| 380 | $7.3183 \times 10^{-42}$ | $8.8013 \times 10^{-40}$ | $1.5364 \times 10^{-39}$ | $1.6172 \times 10^{-40}$ | 850 | $1.0741 \times 10^{-2}$ | $8.0643 \times 10^{-1}$ | $1.0922 \times 10^{-1}$ | $5.3136 \times 10^{-3}$ |
| 390 | $3.3634 \times 10^{-40}$ | $3.9365 \times 10^{-38}$ | $6.1688 \times 10^{-38}$ | $6.3113 \times 10^{-39}$ | 860 | $2.9168 \times 10^{-2}$ | 2.1830 | $2.8766 \times 10^{-1}$ | $1.3875 \times 10^{-2}$ |
| 400 | $1.2977 \times 10^{-38}$ | $1.4807 \times 10^{-36}$ | $2.0929 \times 10^{-36}$ | $2.0829 \times 10^{-37}$ | 870 | $7.7839 \times 10^{-2}$ | 5.8078 | $7.4501 \times 10^{-1}$ | $3.5634 \times 10^{-2}$ |
| 410 | $4.2572 \times 10^{-37}$ | $4.7434 \times 10^{-35}$ | $6.0735 \times 10^{-35}$ | $5.8842 \times 10^{-36}$ | 880 | $2.0426 \times 10^{-1}$ | 1.5195 | 1.8984 | $9.0058 \times 10^{-2}$ |
| 420 | $1.2012 \times 10^{-35}$ | $1.3088 \times 10^{-33}$ | $1.5244 \times 10^{-33}$ | $1.4388 \times 10^{-34}$ | 890 | $5.2731 \times 10^{-1}$ | 3.9115 | 4.7621 | $2.2409 \times 10^{-1}$ |
| 430 | $2.9455 \times 10^{-34}$ | $3.1430 \times 10^{-32}$ | $3.3428 \times 10^{-32}$ | $3.0758 \times 10^{-33}$ | 900 | 1.3400 | 9.9118 | 1.1765 | $5.4928 \times 10^{-1}$ |
| 440 | $6.3373 \times 10^{-33}$ | $6.6304 \times 10^{-31}$ | $6.4625 \times 10^{-31}$ | $5.8012 \times 10^{-32}$ | 910 | 3.3533 | $2.4737 \times 10^{2}$ | 2.8639 | 1.3268 |
| 450 | $1.2067 \times 10^{-31}$ | $1.2393 \times 10^{-29}$ | $1.1107 \times 10^{-29}$ | $9.7334 \times 10^{-31}$ | 920 | 8.2681 | $6.0832 \times 10^{2}$ | 6.8724 | 3.1600 |
| 460 | $2.0494 \times 10^{-30}$ | $2.0684 \times 10^{-28}$ | $1.7100 \times 10^{-28}$ | $1.4639 \times 10^{-29}$ | 930 | 2.0095 | $1.4747 \times 10^{3}$ | $1.6264 \times 10^{2}$ | 7.4232 |
| 470 | $3.1273 \times 10^{-29}$ | $3.1048 \times 10^{-27}$ | $2.3748 \times 10^{-27}$ | $1.9872 \times 10^{-28}$ | 940 | 4.8160 | $3.5254 \times 10^{3}$ | $3.7975 \times 10^{2}$ | 1.7207 |
| 480 | $4.3159 \times 10^{-28}$ | $4.2189 \times 10^{-26}$ | $2.9939 \times 10^{-26}$ | $2.4503 \times 10^{-27}$ | 950 | $1.1387 \times 10^{2}$ | $8.3152 \times 10^{3}$ | $8.7515 \times 10^{2}$ | 3.9375 |
| 490 | $5.4199 \times 10^{-27}$ | $5.2208 \times 10^{-25}$ | $3.4464 \times 10^{-25}$ | $2.7606 \times 10^{-26}$ | 960 | $2.6570 \times 10^{2}$ | $1.9357 \times 10^{4}$ | $1.9914 \times 10^{3}$ | 8.8974 |
| 500 | $6.2278 \times 10^{-26}$ | $5.9162 \times 10^{-24}$ | $3.6422 \times 10^{-24}$ | $2.8568 \times 10^{-25}$ | 970 | $6.1211 \times 10^{2}$ | $4.4492 \times 10^{4}$ | $4.4758 \times 10^{3}$ | 1.9862 |
| 510 | $6.5818 \times 10^{-25}$ | $6.1708 \times 10^{-23}$ | $3.5512 \times 10^{-23}$ | $2.7291 \times 10^{-24}$ | 980 | $1.3927 \times 10^{3}$ | $1.0101 \times 10^{5}$ | $9.9397 \times 10^{3}$ | $4.3814 \times 10^{2}$ |
| 520 | $6.4282 \times 10^{-24}$ | $5.9520 \times 10^{-22}$ | $3.2091 \times 10^{-22}$ | $2.4176 \times 10^{-23}$ | 990 | $3.1308 \times 10^{3}$ | $2.2657 \times 10^{5}$ | $2.1818 \times 10^{4}$ | $9.5545 \times 10^{2}$ |
| 530 | $5.8274 \times 10^{-23}$ | $5.3321 \times 10^{-21}$ | $2.6992 \times 10^{-21}$ | $1.9945 \times 10^{-22}$ | 1000 | $6.9559 \times 10^{3}$ | $5.0231 \times 10^{5}$ | $4.7352 \times 10^{4}$ | $2.0603 \times 10^{3}$ |
| 540 | $4.9233 \times 10^{-22}$ | $4.4545 \times 10^{-20}$ | $2.1214 \times 10^{-20}$ | $1.5383 \times 10^{-21}$ | 1010 | $1.5278 \times 10^{4}$ | $1.1010 \times 10^{6}$ | $1.0164 \times 10^{5}$ | $4.3947 \times 10^{3}$ |
| 550 | $3.8913 \times 10^{-21}$ | $3.4832 \times 10^{-19}$ | $1.5636 \times 10^{-19}$ | $1.1132 \times 10^{-20}$ | 1020 | $3.3188 \times 10^{4}$ | $2.3869 \times 10^{6}$ | $2.1585 \times 10^{5}$ | $9.2751 \times 10^{3}$ |
| 560 | $2.8872 \times 10^{-20}$ | $2.5583 \times 10^{-18}$ | $1.0844 \times 10^{-18}$ | $7.5836 \times 10^{-20}$ | 1030 | $7.1316 \times 10^{4}$ | $5.1190 \times 10^{6}$ | $4.5363 \times 10^{5}$ | $1.9374 \times 10^{4}$ |
| 570 | $2.0177 \times 10^{-19}$ | $1.7706 \times 10^{-17}$ | $7.0993 \times 10^{-18}$ | $4.8790 \times 10^{-19}$ | 1040 | $1.5164 \times 10^{5}$ | $1.0864 \times 10^{7}$ | $9.4371 \times 10^{5}$ | $4.0065 \times 10^{4}$ |
| 580 | $1.3320 \times 10^{-18}$ | $1.1581 \times 10^{-16}$ | $4.3999 \times 10^{-17}$ | $2.9730 \times 10^{-18}$ | 1050 | $3.1915 \times 10^{5}$ | $2.2822 \times 10^{7}$ | $1.9439 \times 10^{6}$ | $8.2045 \times 10^{4}$ |
| 590 | $8.3309 \times 10^{-18}$ | $7.1797 \times 10^{-16}$ | $2.5885 \times 10^{-16}$ | $1.7204 \times 10^{-17}$ | 1060 | $6.6501 \times 10^{5}$ | $4.7468 \times 10^{7}$ | $3.9657 \times 10^{6}$ | $1.6642 \times 10^{5}$ |
| 600 | $4.9493 \times 10^{-17}$ | $4.2296 \times 10^{-15}$ | $1.4493 \times 10^{-15}$ | $9.4786 \times 10^{-17}$ | 1070 | $1.3723 \times 10^{6}$ | $9.7778 \times 10^{7}$ | $8.0147 \times 10^{6}$ | $3.3443 \times 10^{5}$ |
| 610 | $2.7998 \times 10^{-16}$ | $2.3735 \times 10^{-14}$ | $7.7408 \times 10^{-15}$ | $4.9838 \times 10^{-16}$ | 1080 | $2.8050 \times 10^{6}$ | $1.9952 \times 10^{8}$ | $1.6050 \times 10^{7}$ | $6.6602 \times 10^{5}$ |
| 620 | $1.5116 \times 10^{-15}$ | $1.2717 \times 10^{-13}$ | $3.9527 \times 10^{-14}$ | $2.5063 \times 10^{-15}$ | 1090 | $5.6807 \times 10^{6}$ | $4.0338 \times 10^{8}$ | $3.1856 \times 10^{7}$ | $1.3147 \times 10^{6}$ |
| 630 | $7.8064 \times 10^{-15}$ | $6.5188 \times 10^{-13}$ | $1.9337 \times 10^{-13}$ | $1.2080 \times 10^{-14}$ | 1100 | $1.1401 \times 10^{7}$ | $8.0827 \times 10^{8}$ | $6.2680 \times 10^{7}$ | $2.5730 \times 10^{6}$ |
| 640 | $3.8637 \times 10^{-14}$ | $3.2038 \times 10^{-12}$ | $9.0811 \times 10^{-13}$ | $5.5911 \times 10^{-14}$ | 1110 | $2.2683 \times 10^{7}$ | $1.6054 \times 10^{9}$ | $1.2228 \times 10^{8}$ | $4.9933 \times 10^{6}$ |
| 650 | $1.8363 \times 10^{-13}$ | $1.5124 \times 10^{-11}$ | $4.1011 \times 10^{-12}$ | $2.4895 \times 10^{-13}$ | 1120 | $4.4741 \times 10^{7}$ | $3.1617 \times 10^{9}$ | $2.3660 \times 10^{8}$ | $9.6112 \times 10^{6}$ |
| 660 | $8.3952 \times 10^{-13}$ | $6.8697 \times 10^{-11}$ | $1.7842 \times 10^{-11}$ | $1.0682 \times 10^{-12}$ | 1130 | $8.7513 \times 10^{7}$ | $6.1748 \times 10^{9}$ | $4.5410 \times 10^{8}$ | $1.8352 \times 10^{7}$ |
| 670 | $3.6983 \times 10^{-12}$ | $3.0075 \times 10^{-10}$ | $7.4898 \times 10^{-11}$ | $4.4242 \times 10^{-12}$ | 1140 | $1.6978 \times 10^{8}$ | $1.1962 \times 10^{10}$ | $8.6467 \times 10^{8}$ | $3.4771 \times 10^{7}$ |
| 680 | $1.5724 \times 10^{-11}$ | $1.2711 \times 10^{-9}$ | $3.0383 \times 10^{-10}$ | $1.7713 \times 10^{-11}$ | 1150 | $3.2677 \times 10^{8}$ | $2.2988 \times 10^{10}$ | $1.6338 \times 10^{9}$ | $6.5376 \times 10^{7}$ |
| 690 | $6.4614 \times 10^{-11}$ | $5.1933 \times 10^{-9}$ | $1.1928 \times 10^{-9}$ | $6.8654 \times 10^{-11}$ | 1160 | $6.2402 \times 10^{8}$ | $4.3837 \times 10^{10}$ | $3.0640 \times 10^{9}$ | $1.2201 \times 10^{8}$ |
| 700 | $2.5699 \times 10^{-10}$ | $2.0542 \times 10^{-8}$ | $4.5377 \times 10^{-9}$ | $2.5794 \times 10^{-10}$ | 1170 | $1.1826 \times 10^{9}$ | $8.2962 \times 10^{10}$ | $5.7040 \times 10^{9}$ | $2.2605 \times 10^{8}$ |
| 710 | $9.9067 \times 10^{-10}$ | $7.8767 \times 10^{-8}$ | $1.6750 \times 10^{-8}$ | $9.4059 \times 10^{-10}$ | 1180 | $2.2247 \times 10^{9}$ | $1.5585 \times 10^{11}$ | $1.0542 \times 10^{10}$ | $4.1583 \times 10^{8}$ |
| 720 | $3.7058 \times 10^{-9}$ | $2.9314 \times 10^{-7}$ | $6.0066 \times 10^{-8}$ | $3.3331 \times 10^{-9}$ | 1190 | $4.1546 \times 10^{9}$ | $2.9066 \times 10^{11}$ | $1.9349 \times 10^{10}$ | $7.5965 \times 10^{8}$ |
| 730 | $1.3468 \times 10^{-8}$ | $1.0601 \times 10^{-6}$ | $2.0949 \times 10^{-7}$ | $1.1490 \times 10^{-8}$ | 1200 | $7.7038 \times 10^{9}$ | $5.3825 \times 10^{11}$ | $3.5268 \times 10^{10}$ | $1.3783 \times 10^{9}$ |

## 6. AutoMeKin reaction network details.

To construct the reaction network, we first generated a preliminary low-level (LL) network using the AutoMeKin package. The reaction mechanism was initially explored via accelerated molecular dynamics, with geometry optimizations performed at the semi-empirical PM7 level of theory using MOPAC. During this stage, all generated structures were screened to remove duplicates, resulting in the elimination of 214 redundant transition state (TS) structures. The final LL network consisted of 2,611 TSs, 1,720 minima, and 2,057 dissociation products.

Subsequently, a higher-level (HL) network was constructed by re-optimizing the LL TS geometries using a more accurate level of theory by means of DFT methods. This HL refinement included a second round of structural deduplication, which led to the removal of an additional 88 TS structures. To focus on kinetically relevant pathways and avoid computationally expensive treatment of high-energy processes, an energy cutoff of 100 kcal/mol was applied. Only structures within this threshold were retained for re-optimization. The final HL reaction network comprises 1,708 TSs, 1,333 minima, and 119 dissociation products.

## 7. Kinetic Monte Carlo simulations details.

The kinetic Monte Carlo (kMC) simulations were performed using the Pilgrim program to model the time-dependent evolution of a chemical system involving two competing unimolecular reaction pathways. Simulations were carried out under isothermal and isobaric conditions, with the temperature and pressure explicitly defined. The kMC algorithm employs a residence-time (rejection-free) approach, where reaction events are selected probabilistically based on their computed rate constants and the instantaneous species populations. The simulation was initialized with $1.00 \times 10^{20}$ molecules in a fixed volume of 1.00 mL, assuming a well-mixed environment and no diffusion limitations. This setup enables a realistic stochastic modelling of the competition between the two pathways and their kinetic contributions over time.
